# Resveratrol and curcumin as natural alternatives to monensin for preventing in vitro induced ruminal acidosis in buffalo bulls

**DOI:** 10.1007/s11250-025-04632-z

**Published:** 2025-09-22

**Authors:** Eman A. Elwakeel, Mariam G. Ahmed, Hanan B. El-sawy, Samir Z. El-Zarkouny, Adham A. Al-Sagheer

**Affiliations:** 1 Department of Animal and Fish Production, Faculty of Agriculture (El-Shatby), Alexandria University, Alexandria, 21545 Egypt; 2 Animal Production Research Institute, Agriculture Research Center, Nadi El-Said, Giza, 11622 Egypt; 3 Department of Nutrition and Clinical Nutrition, Faculty of Veterinary Medicine Kafrelsheikh University, Kafrelsheikh, 33516 Egypt; 4 Animal Production Department, Faculty of Agriculture, Zagazig University, Zagazig, 44511 Egypt

**Keywords:** Resveratrol, Curcumin, Lactic acid, Ruminal acidosis, Volatile fatty acids

## Abstract

This study included three experiments using a semi-automated in vitro gas production technique to investigate the potential of resveratrol (RES) and curcumin (CUR) as natural alternatives to monensin (MON) for preventing ruminal acidosis. In Experiment 1, different concentrations (100, 200, and 400 µg/mL) of RES, CUR, a combination of both (MIX), MON (12 µg/mL), and a control group were evaluated for their ability to counteract ruminal acidosis. Acidosis was induced by incubating glucose (0.1 g/mL) without buffer for 6 h to simulate a highly fermentable, unbuffered ruminal environment. In Experiment 2, RES (200 µg/mL), CUR (200 µg/mL), MIX (200 µg/mL), and MON were compared when buffer was added, and fermentation was extended to 24 h using the same substrate as in Experiment 1. Experiment 3 examined the effect of RES (200 µg/mL) on acidosis in vitro using corn, barley, or wheat as substrates, and compared it to MON. In Experiment 1, pH increased significantly (*P* < 0.05) and gas production decreased (*P* < 0.01) with the addition of RES and MIX at 200 and 400 g/mL as well as MON at 12 µg/mL, whereas CUR had no effect on pH (*P* > 0.05). In Experiment 2, both RES and MON increased pH (*P* < 0.01) and reduced (*P* < 0.01) lactic acid concentration. MON and RES increased (*P* < 0.01) propionic acid and reduced (*P* < 0.01) the acetate to propionate ratio. While MON significantly reduced total volatile fatty acid (VFA) concentrations (*P* < 0.01) compared to the control, RES had no significant effect. In Experiment 3, when corn was used as a substrate, RES was found to significantly increase (*P* < 0.01) pH and decrease lactic acid levels in comparison to barley or wheat. MON improved (*P* < 0.01) propionate levels and decreased (*P* < 0.01) the ratio of acetate to propionate. Both RES and MON did not affect the total VFA. In conclusion, RES shows promise as a natural alternative to MON for preventing rumen acidosis, particularly when corn is used as a basal diet. The findings indicate that RES can effectively increase ruminal pH and reduce lactic acid levels, which are key factors in mitigating acidosis.

## Introduction

Ruminal acidosis is the most common metabolic disorder that affects ruminants, such as cattle, buffalo, sheep, and goats (Khaskheli et al. 2020; Voulgarakis et al. 2023). The primary cause of ruminal acidosis is the consumption of large amounts of highly fermentable carbohydrates, such as grains and concentrate (Monteiro and Faciola 2020), that lead to an increase in organic acid production (VFAs and lactic acid) in the rumen, which causes a rapid drop in pH levels, leading to ruminal acidosis (Mao and Wang 2025). The drop in ruminal pH can alter the microbial population in the rumen, leading to an imbalance between acid producing and acid-utilizing bacteria (Elmhadi et al. 2022; Mao et al. 2013) leading to a decrease in feed intake, poor performance, and even death (Monteiro and Faciola 2020). The severity of ruminal acidosis can range from mild (subacute acidosis) to severe (acute acidosis), depending on the duration and intensity of acidification. Subacute acidosis may result in decreased feed intake and milk production, while acute acidosis can lead to dehydration, diarrhea, laminitis, liver abscesses, and even death (Kofler et al. 2023; Udainiya et al. 2024).

Monensin (MON) is a feed additive that is commonly used in the livestock industry to improve feed efficiency and promote growth in ruminants (Maier et al. 2024). Several researches showed that MON was able to modulate rumen fermentation (Prusty et al. 2024), improved animal production such as milk yield (Kozerski et al. 2017), body weight (Neumann et al. 2018), and control digestive disorder and animal diseases (Prusty et al. 2024). One of the key benefits of MON is its ability to reduce the incidence of ruminal acidosis (Elmhadi et al. 2022; Televicius et al. 2019). MON has been approved as an effective tool for reducing the risk of ruminal acidosis by altering the microbial population and maintaining a more stable pH in the rumen (Cobos et al. 2011). In sheep, MON supplementation showed a prophylactic effect by decreasing the rate of ruminal pH decline and occasionally reducing ruminal acidosis (Reis et al. 2018). However, using antibiotics as feed additives has been banned in the European Union because of their residues in human food and, more importantly, because they can cause bacterial resistance (Shahid et al. 2021). Therefore, studies have shifted to another safer modifier for rumen fermentation, such as plant extracts and their secondary metabolites (Ahmed et al. 2024; Al-Sagheer et al. 2018). In the post-antibiotic era, several natural additives have been proposed as alternatives to monensin. Phytochemicals, in particular, have gained attention for their ability to modulate rumen fermentation and microbial populations without contributing to antimicrobial resistance. For example, Ahmed et al. (2022) reported that grape seed extract was effective in controlling ruminal acidosis in vitro. Similarly, Rivera-Chacon et al. (2022) reported that dietary supplementation with a phytogenic blend, comprising thymol, L-menthol, eugenol, *Mentha arvensis* oil, and *Syzygium aromaticum* powder in high-concentrate diets for dairy cows altered the rumen microbial ecosystem and reduced the risk of ruminal acidosis.

Resveratrol (RES) is a natural phenol that is found in the seeds and skin of red grapes and has been shown to have antimicrobial activity against harmful bacteria (Bejenaru et al. 2024). Mechanistically, RES has been shown in vitro to reduce methane production by suppressing *Methanobrevibacter* populations and increase propionate proportion under high-concentrate diets, thereby shifting fermentation away from lactate accumulation (Ma et al. 2020). Furthermore, feeding RES to ruminants has been reported to enhance populations of fibrolytic bacteria such as *Fibrobacter succinogenes*, *Ruminococcus albus*, and *Butyrivibrio fibrisolvens*, while decreasing protozoa and methanogens, changes that align with improved ruminal pH stability and reduced acid load (Meng et al. 2023). In vivo, dietary RES increased ruminal pH from 6.55 to 6.73 in ewes, elevated total VFA and acetate, and modified microbial-metabolite profiles linked to health and resilience to metabolic stress (Li et al. 2024). Curcumin (CUR), a polyphenolic compound extracted from turmeric, exhibits antimicrobial, antioxidant, and anti-inflammatory properties relevant to rumen health (Tian et al. 2023). Numerous studies show plant polyphenols could enhance the activity of lactate-utilizing bacteria that convert lactic acid into less harmful compounds, thereby preventing the accumulation of lactic acid and maintaining a stable ruminal pH (Ahmed et al. 2022; Balcells et al. 2012; De Nardi et al. 2016).

To our knowledge, no research is available on the effect of RES and CUR on ruminal acidosis. Our hypothesis is that RES, CUR, and their mixture could inhibit rumen acidosis in vitro. Hence, the current study investigated the effects of RES and CUR as natural alternatives to MON on in vitro ruminal acidosis, emphasizing ruminal pH, gas production, lactic acid concentration, and volatile fatty acids (VFA). The study also aimed to compare the effectiveness of RES and CUR with MON in preventing ruminal acidosis when different substrates (corn, barley, and wheat) were used. The outcome of this study can provide important insights into their potential as natural solutions for ruminal acidosis prevention in ruminants.

## Materials and methods

### Animal donor and inoculum preparation

Three male Egyptian buffaloes, with an average weight of 450 ± 50 kg, were slaughtered in an abattoir managed by the Faculty of Agriculture (El-Shatby) in Alexandria city, Egypt, in order to collect the rumen fluid. The experimental protocols applied in this study were performed in accordance with the guidelines for animal welfare of the Animal and Fish Production Department, Faculty of Agriculture (El-Shatby), Alexandria University, Egypt, and were approved by Ethics Committee of the Local Experimental Animals Care with the assigned approval number Alex. Agri. 082305302. The study also was carried out in agreement with ARRIVE recommendations (Du Sert et al. 2020). The collection of rumen content from slaughtered buffaloes is compliant with animal welfare regulations and is a cost-effective technique that eliminates the need for surgical cannulas in living animals (Mutimura et al. 2013). Additionally, several previous in vitro fermentation studies recommended and validated the alternative use of rumen fluid from slaughtered animals (Ahmed et al. 2023; Wang et al. 2017). The animals that were slaughtered (following the halal slaughtering method) were fed traditional feedlot diet, which was composed of 30% Berseem hay and 70% concentrate mixture (16% CP). Following a 15-minute of slaughter, the rumen was slashed with a scalpel and its fluids were extracted from multiple locations. The contents of the rumen were quickly filtered using four layers of gauze, deposited in preheated thermos containers to maintain anaerobic conditions and a temperature of 39° C, and transported directly to the laboratory. Before the incubation process, the rumen juice was thoroughly mixed and filtered with four layers of cheesecloth. In each laboratory experiment, the starting pH level of the rumen juice was recorded using a portable pH meter (model GLP21; CRISON, Barcelona, Spain).

### Experiment 1 (screening assay)

#### Treatments, experimental design, and incubation system

The effects of RES and CUR were tested individually, and their mixture on pH and gas production were compared to those of control (CON; negative control) and MON (positive control) when acidosis was induced in vitro. Stock solutions of RES, CUR, and a combination of both were prepared using dimethyl sulfoxide (SD Fine-Chem Limited Company, Mumbai, India) for obtaining concentrations of 10, 20, and 40 mg/mL. Just before inoculation, 450 µl of stock solutions of RES, CUR, and MIX were added to 45 ml of rumen fluid to provide 100, 200, and 400 µg /mL. Also, a stock solution of MON (Rumensin^®^, Elanco, Itapira, Brazil) was prepared in dimethyl sulfoxide to provide 12 µg /mL when 450 µl of that stock solution was pipetted into 45 mL of rumen inoculum according to Ala et al. (2020). Treatments were evaluated using the method provided by Hutton et al. (2010), which depend on measuring the pH values after 6 h of fermentation without the use of a buffer. The reason for not adding buffer in this experiment is to prevent an excessively slow pH decline and to compare the pH levels of various treatments.

To induce acidosis, 4.5 g of glucose and 0.45 g of berseem hay were weighed and placed in a 120-ml serum bottles as a negative control, following our laboratory protocol. Subsequently, the bottles (6 replicates/ treatment) were subjected to incubation at 39 °C for 6 h. Next, a volume of 45 mL of ruminal inoculum was placed into the serum bottles, followed by the addition of 450 µl of the previously produced treatment solutions into the 45 mL of rumen fluid. The pH of the mixed rumen fluid was initially determined, and 450 µl of dimethyl sulfoxide was added into the control bottles shortly before the beginning of the incubation procedure. The bottles were subjected to CO_2_ gas, followed by sealing with rubber stoppers and aluminum crimps. To achieve a pressure of zero in the headspace of the bottles, a 23G needle was inserted into the stopper. Subsequently, all of the bottles were moved to the incubator and subjected to incubation at a temperature of 39 °C for a period of 6 h.

#### Sample collection

At 2, 4, and 6 h of incubation, the gas pressure inside the bottles was measured by placing a 23G needle into the headspace. This was accomplished with the use of a pressure transducer and data logger (GN200, Sao Paulo, Brazil). To prevent temperature-related pressure errors, the bottles were put in a water bath set at a temperature of 39 °C while conducting gas pressure measures, as recommended by Bettelheim et al. (2012). Following each measurement of gas pressure, a 23G needle was used to puncture the rubber stopper of the bottles in order to minimize the gas pressure. The pH data were taken 6-hour after the rubber stoppers were removed.

### Experiment 2 (protection assay)

#### Treatments, experimental design, and incubation conditions

This experiment measured lactic acid and VFA production to compare the effects of RES, CUR, and their mixture to MON on acidosis. The treatments were CON (no supplementation), RES (200 µg /mL), CUR (200 µg /mL), and a mixture of RES and CUR (200 µg /mL; 100 µg /mL each). The second experiment was conducted under incubation conditions that were similar to those of the first experiment, with a couple of exceptions: 1- Fermentation was prolonged from six to twenty-four hours 2-Bufer was added to the assay because the buildup of organic acids over a 24-hour period could cause the pH value to decline markedly. The rumen fluid (22.5 mL) and McDougall’s buffer (22.5 mL; (McDougall 1948) were dispensed into 120 mL serum bottles (6 bottles/ treatment). Similar to the first experiment, glucose concentrations (acidosis stimulants) were employed.

#### Sample collection

During an incubation period of 2, 4, 6, 9, 12, and 24 h, the cumulative gas production was measured. At 6, 12, and 24 h of incubation, a 23G needle was injected into the rubber stopper of the container to withdraw 1.5 ml of liquid. About 1 mL was used to estimate the pH value. The other half was used for VFA and lactic acid analysis by transferring 0.5 mL of samples to 1.5 ml Eppendorf tubes. After acidification with 100µL of 25% (w/v) meta-phosphoric acid, the samples were stored at − 20 °C for future analysis. Upon thawing, the samples were centrifuged at 30,000 ×g (JA-17 rotor) for 20 min, and the supernatant was utilized to determine the levels of lactic acid and VFA. Supernatant (250 µL) was used for Lactic acid measurement spectrophotometrically according the method developed by Borshchevskaya et al. (2016). To analyse the VFA content, 250 µL of the remaining supernatants were transferred to GC vials, and 1 µL of the sample was injected into a gas chromatography (GC Thermo TRACE 1300) equipped with a capillary column (TR-FFAP 30 m × 0.5 μm × 0.53 mmI D). The oven temperature was raised to 200 °C at 10 °C per min, while the flame ionization detectors and injection temperatures were adjusted to 250 °C and 220 °C, respectively. The carrier gas used in this experiment was nitrogen, which was supplied at a flow rate of 7 ml/min. Hydrogen and make-up gases were also utilized, with flow rates of 40 ml/min and 35 ml/min, respectively. The total run time was 10 min, and a standard with known VFA concentrations was used for calibration.

### Experiment 3 (grain assay)

This experiment aimed to examine the effect of MON and RES on pH, VFA, and lactic acid concentrations when corn, wheat, or barley grains were utilized as substrates. The treatments were arranged in a 3 × 3 factorial design, including three substrates (corn, wheat, and barley) and three treatments (CON, MON, and RES). The grains were finely milled to pass through a 1 mm screen, 4.5 g of powdered grains were carefully placed into 120 mL serum bottles (6 bottles/ treatment) to induce rumen acidosis, following the protocol of Dennis et al. (1981). Sample collection, the concentrations of RES and MON, and procedure were comparable to those in Experiment 2.

#### Statistical analyses

Data were analyzed by ANOVA for complete block designs, with replicates or different runs as the blocking factor, by using the MIXED procedure of SAS (version 9.1, SAS Inst., INC., Cary, NC). Blocks and treatments were included in the model effects, in addition to all possible interactions. The main effects of treatment (CON, MON, RES, CUR and MIX) time (6, 12, 24 h), and their interactions were included in the model. For EXP.1, observations were compared to CON or MON. For EXP.1 through 3, for all observations that were collected over time, repeated measures were applied, with time and all interactions of time with treatments included. Prior to performing ANOVA, data were assessed for normality using the Shapiro–Wilk test (*p* > 0.05) and Q–Q plots. Homogeneity of variances was evaluated using Levene’s test. All data met the assumptions of normality and homoscedasticity, confirming the suitability of ANOVA using the MIXED procedure.

## Results

### Experiment 1

Table 1 shows the effect of RES, CUR, and their MIX at different concentrations on gas production and pH. Treatments had a significant (*P* < 0.0001) impact on gas production and pH. The higher concentrations of RES (200, 400 µg /L) reduced gas production and increased pH compared to CON. However, the pH of RES 200 was comparable to that of MON, but the pH of RES 400 was higher than MON. The lower concentration of CUR (100 µg /L) increased the GP and reduced the pH compared to the CON. The 200 CUR had no effect on gas production or pH compared to CON. However, CUR 400 µg /L reduced gas production compared to the CON without having an effect on pH. MIX (200,400 µg /mL) and MON increased pH (*P* < 0.05) compared to CON. However, MIX (200, 400 µg /mL), and MON reduced (*P* < 0.01) gas production.

**Table 1:** Effect of Resveratrol (RES), Curcumine (CUR) and mixture of Resveratrol and Curcumine (MIX) at different levels (100, 200, 400 µg/mL) on gas production (GP; Kpa) and pH compared to control (CON) and Monensin (MON) at 6 h fermentation using hay (0.01 g/mL rumen fluid) and glucose (0.1 g/mL rumen fluid) as a substrate (EXP.1)

Treatments	Amount (µg/ml)	Gas Production (KPa)	pH
CON	0	121.0‡	5.13‡
MON	12	97.7*	5.72*
RES (µg/mL)			
	100	112.1†	5.34‡
	200	106.6*	5.58*
	400	78.60*‡	6.12*‡
CUR (µg/mL)			
	100	137.1*‡	4.91‡
	200	120.4‡	5.19‡
	400	104.9*	5.02‡
MIX (µg/mL)			
	100	125.4‡	5.22‡
	200	110.0*‡	5.43*‡
	400	88.41*	5.84*
SEM^4^		3.423	0.138
p-value		< 0.0001	< 0.0001

*Means within column are different (*P* < 0.05) from the control

‡ Means within column are different (*P* < 0.05) from monensin

^4^ SEM = standard error of the mean. The reported values represent the average obtained from six replicates per treatment

### Experiment 2

Based on the results of experiment 1, a concentration of 200 µg /mL of both RES and MIX (a mixture of CUR and RES) was chosen for this experiment because they increased pH compared to CON and at the same time increased gas production compared to MON. Tables 2 and 3 represent the effects of RES, CUR, and their mix at 200 µg /L on GP and pH, respectively. There was no interaction (*P* = 0.531) between treatment and time on GP. Treatment and time had a significant (*P* < 0.001) impact on GP. There was no effect of RES and CUR on gas production. In contrast, MIX and MON produced less gas than the CON.

**Table 2:** Effect of Monensin (MON; 12 µg/ml), Resveratrol (RES; 200 µg/ml), Curcumin (CUR; 200 µg/ml) and mixture of Resveratrol and Curcumin (MIX; 100 µg/ml each) compared to control (CON; no additives) on gas production (EXP.2)

Treatments	Gas production (KPa)					
	3 h	6 h	9 h	12 h	24 h	Overall
CON	50.81^a^	85.75^b^	133.2^a^	149.9^a^	156.8^a^	115.30^a^
MON	26.16^b^	52.15^d^	84.9^b^	119.4^b^	132.3^b^	82.97^b^
RES	36.46^ab^	77.8^bc^	123.4^a^	146.7^a^	160^a^	108.83^a^
CUR	44.47^ab^	110.7^a^	139.9^a^	147.9^a^	153.1^ab^	119.23^a^
MIX	26.84^b^	61.15^cd^	101.5^b^	116.5^b^	132.29^b^	87.83^b^
SEM	2.35	4.95	4.97	3.61	2.94	3.57

Treatment effect *P* < 0.0001, Time *P* < 0.001, TRT × Time *P* = 0.531

^a−d^ Means in the same column with different superscripts differ (*P* < 0.05)

SEM = standard error of the mean. The reported values represent the average obtained from six replicates per treatment

**Table 3:** Effect of Monensin (MON; 12 µg/ml), Resveratrol (RES; 200 µg/ml), Curcumin (CUR; 200 µg/ml) and mixture of Resveratrol and Curcumin (MIX; 100 µg/ml each) compared to control (CON; no additives) on ruminal pH (EXP.2)

Treatments	Time			Overall
	6 h	12 h	24 h	
CON	6.32^dA^	4.31^cB^	4.10^dC^	4.91^c^
MON	6.84^aA^	5.12^aB^	4.51^aC^	5.49^a^
RES	6.54^cA^	4.59^bB^	4.33^bC^	5.15^b^
CUR	5.12^eA^	4.29^cB^	4.21^cB^	4.54^d^
MIX	6.66^bA^	4.56^bB^	4.37^bC^	5.2^b^
SEM	0.033	0.016	0.014	0.017

^a−e^ Means in the same column with different superscripts differ (*P* < 0.05)

^A−C^ Means in the same row with different superscripts differ (*P* < 0.05)

Effect of treatment, time, and treatment × time were all significant at *P* < 0.0001

^3^ SEM = standard error of the mean. *n* = 6 replicates / treatment

There was an effect of treatment, time, and their interaction on pH. The cause of this interaction is that the pH declined at 12 h and more at 24 h for all treatments except CUR, which caused the pH to decline at 12 h without further decline at 24 h. RES and MIX increased the overall values of pH (5.15 and 5.2, respectively) compared to the CON (4.91), with MON achieving the highest value (5.49). On the other hand, CUR caused the lowest pH (4.54) compared to CON (4.91). As shown in Table 4, treatments, time, and their interaction affected the molar concentration of lactic acid (*P* < 0.01). CUR treatment produced the highest lactic acid concentration (98.57 mM), followed by CON (75.06 mM). However, MON, RES, and MIX produced the lowest (*P* < 0.001) lactic acid concentration, which was 55.94 mM, 58.93 mM, and 60.54 mM, respectively.

**Table 4:** Effect of Monensin (MON; 12 µg/ml), Resveratrol (RES; 200 µg/ml), Curcumin (CUR; 200 µg/ml) and mixture of Resveratrol and Curcumin (MIX; 100 µg/ml each) compared to control (CON; no additives) on lactic acid concentration (mM; EXP.2)

Time	Treatment					
	CON	MON	RES	CUR	MIX	SEM
6 h	21.45^bC^	6.91^cC^	8.15^cC^	71.43^aC^	13.56^cC^	4.85
12 h	96.82^bB^	75.2^cB^	78.2^cB^	105.8^aB^	78.87^cB^	2.44
24 h	103.9^bA^	85.71^cA^	88.7^cA^	118.5^aA^	89.19^cA^	2.49
Overall	75.06^b^	55.94^c^	58.33^c^	98.57^a^	60.54^c^	1.30

The effect of treatments and time and their interaction were significant (*P* < 0.001)

a-d Means in the same row with different superscripts differ (*P* < 0.05)

A-C Means in the same column with different superscripts differ (*P* < 0.05)

SEM = standard error of the mean. *n* = 6 replicates / treatment

Data in Table 5 represents the overall effects of RES, CUR, and MIX on VFA over 6, 12, and 24 h compared to CON and MON. The effects of treatment (*P* < 0.05) and time (*P* < 0.001) were significant, however, no interaction was detected between treatments and time (*P* > 0.05). As presented in Table 5, treatment with CUR (59.78 mM) and RES (57.59 mM) did not affect total VFA concentration compared to CON (60.91 mM). However, treatments with MON and MIX reduced the VFA molar concentration (*P* < 0.01) from 60.91 mM in the CON to 50.20 mM and 53.37 mM, respectively.

**Table 5:** Effect of Monensin (MON; 12 µg/ml), Resveratrol (RES; 200 µg/ml), Curcumin (CUR; 200 µg/ml) and mixture of Resveratrol and Curcumin (MIX; 100 µg/ml each) compared to control (CON; no additives) on VFA using hay (0.01 g/mL rumen fluid) and glucose (0.1 g/mL rumen fluid) as a substrate (EXP.2)^1^

	Treatment						
	CON	MON	RES	CUR	MIX	SEM^3^	*P*-value
Total VFA (mM)	60.91^a^	50.20^b^	57.59^a^	59.78^a^	53.37^b^	2.05	0.008
Acetate, %	73.05^a^	71.91^a^	70.91^b^	72.98^a^	72.62^a^	0.52	0.02
Propionate, %	13.29^b^	14.54^a^	15.07^a^	12.71^b^	14.54^a^	0.27	< 0.0001
Butyrate, %	10.45^b^	11.34^a^	11.76^a^	10.63^b^	11.63^a^	0.26	0.006
Iso-valerate, %	1.23^b^	1.52^a^	1.68^a^	1.52^a^	1.79^a^	0.06	< 0.0001
Valerate, %	0.63^b^	0.69^b^	0.84^a^	0.89^a^	0.89^a^	0.024	< 0.0001
A/P	5.50^a^	4.95^b^	4.69^b^	5.74^a^	4.99^b^	0.14	0.0002

^a−b^ Means in the same row with different superscripts differ (*P* < 0.05)

1Data on this table represent average across time (6, 12, 24 h), effect of time was significant for all parameters (*P* < 0.0001), no interaction detected between treatment and time except for valerate (*P* < 0.05)

^3^ SEM = standard error of the mean. *n* = 6 replicates / treatment

The lowest acetate molar concentration (70.91 mM) was observed with (*P* < 0.05) RES addition compared to CON (73.05 mM), MON (71.91 mM), CUR (72.98 mM), and MIX (72.62 mM). On the other hand, the highest propionate molar concentration (*P* < 0.01) was observed with MON (14.54 mM), RES (15.07mM) and the MIX (14.54) compared to CON (13.29 mM) and CUR (12.71mM). Similarly, butyrate concentration was highest (*P* < 0.01) with RES (11.76 mM), MON (11.34 mM), and MIX (11.63 mM) treatments compared to CON (10.45 mM) and CUR (10.63 mM). The highest valerate molar concentration (*P* < 0.01) was observed with RES (0.84 mM), CUR (0.89 mM), and MIX (0.89 mM) compared to MON (0.69 mM) and CON (0.63 mM). The same trend with valerate was observed with isovalerate molare concentration (*P* < 0.01) for the same treatments (Table 5). The lowest (*P* < 0.05) acetate/propionate ratio was recorded with RES (4.69) and MON (4.96) compared to CON (5.32), CUR (5.79), and MIX (5.14).

### Experiment 3

Table 6 shows the effect of MON and RES on pH values, lactic acid concentration and cumulated gas production when corn, wheat or barley were used as substrates. The effects of treatments were significant on pH values, lactic acid concentrations, and accumulative gas production (*P* < 0.001). MON and RES increased overall rumen pH values (4.45 and 4.37, respectively) compared to CON (4.75) and reduced lactic acid concentration from 82.8 mM (CON) to 55.7 and 64.4 mM, respectively. However, RES did not affect gas production similar to MON did compared to CON where MON reduced gas production from 145.5 (CON) to 142.6 kpa.

**Table 6:** Effect of Monensin (12 µg/ml) and Resveratrol (200 µg/ml) on pH, lactic acid concentration and cumulated gas production (EXP.3)

Substrate	Treatment^1^	pH			Overall pH	Lactic acid (mM)	Cumulated GP^2^ (KPa)
		6 h	12 h	24 h			
Corn							
	CON	5.37^b^	4.94^b^	4.35^dc^	4.89^c^	27.51^e^	156.1^a^
	MON	5.60^a^	5.02^a^	4.46^b^	5.03^a^	18.40^f^	143.6^bc^
	RES	5.61^a^	5.03^a^	4.47^b^	5.03^a^	18.23^f^	156.5^a^
Wheat							
	CON	5.28^bc^	4.74^d^	4.26^e^	4.76^d^	93.68^b^	132.7^a^
	MON	5.37^b^	4.87^c^	4.58^a^	4.94^b^	54.52^d^	143.7^bc^
	RES	5.37^b^	4.83^c^	4.41^bc^	4.87^c^	81.57^c^	154.1^a^
Barley							
	CON	5.13^d^	4.56^f^	4.11^f^	4.60^f^	127.34^a^	147.6^b^
	MON	5.14^d^	4.63^e^	4.30^ed^	4.69^e^	87.46^b^	140.6^c^
	RES	5.17^cd^	4.57^ef^	4.24^e^	4.66^e^	93.53^b^	141.7^c^
SEM ^3^		0.015	0.016	0.012	0.014	2.40	1.8
Substrate effect							
Corn		5.53^a^	4.99^c^	4.43^a^	4.98^a^	23.53^c^	152.1^a^
Wheat		5.35^b^	4.81^b^	4.41^a^	4.86^b^	76.59^b^	143.5^b^
Barley		5.14^c^	5.59^a^	4.22^b^	4.65^c^	102.78^a^	143.3^b^
Treatment effect							
	CON	5.26^b^	4.75^b^	4.24^b^	4.75^c^	82.8^a^	145.5^a^
	MON	5.37^a^	4.84^a^	4.45^a^	4.88^a^	55.7^c^	142.6^a^
	RES	5.38^a^	4.81^a^	4.37^a^	4.85^b^	64.4^b^	150.8^b^
SEM^3^		0.011	0.008	0.018	0.011	2.03	1.04

CON = control; MON = monensin; RES = resveratrol. ^2^GP= gas production.^3^ SEM = standard error of the mean. ^a-c^ Means within the same column for each substrate or effect with different superscripts differ (*P* < 0.05). Effect of treatments, substrates, and their interaction were significant (*P* < 0.01) for all parameters. Effect of time and its interaction with substrate (substrate × time) or treatment (treatment × time) were significant (*P* < 0.01) for all parameters. Interaction was detected (*P* < 0.01) between treatment, substrate and time for only pH. No interaction was detected between treatment, substrate and time for lactic acid or cumulated gas production (*P* > 0.05). ^3^ SEM = standard error of the mean. *n* = 6 replicates / treatment

Substrate effects were significant (*P* < 0.001) on overall pH, lactic acid concentrations, and cumulated gas production. When corn was used as substrate, it increased pH (4.98), followed by wheat (4.86) and barley (4.65), which caused the lowest pH value and at the same time produced the highest lactic acid concentration (102.8 mM), while corn produced the lowest lactic acid concentration (23.53 mM). Corn substrate caused the highest gas production (152.1 kpa) compared to wheat (143.5 kpa) and barley (143.3 kpa). An interaction was detected (*P* < 0.01) between treatment and substrate on pH values, lactic acid concentration and cumulated gas production.

The effect of time was significant on pH values, lactic acid concentrations, and accumulative gas production (*P* < 0.001). Also, an interaction was detected (*P* < 0.01) between treatment × time and substrate × time for pH values, lactic acid concentration, and accumulative gas production. No interaction was detected between treatment × substrate × time on lactic acid and cumulative gas production. In contrast, an interaction was observed between treatments × substrates × time on pH values.

The effects of MON and RES on the molar proportion of VFA, total fatty acid concentration, and acetate-to-propionate ratio are presented in Table 7. The substrate and treatment effects were significant (*P* < 0.001) for acetate, propionate, butyric, isovalerate, valerate, and the A/P ratio. However, no effect of substrate or treatment was observed on total VFA concentration. An interaction was detected between treatment and substrate on propionate, butyric, isovaleric, valeric, and A/P ratios. In contrast, no interaction (*P* > 0.05) was detected between treatment and substrate on acetate and total VFA concentration. The effects of time and either its interaction with substrate or treatment were not significant (*P* > 0.05) on individual VFA, total VFA, or the A/P ratio. No interaction (*P* > 0.05) was observed between time, substrate, and treatment on individual VFA, total VFA, or A/P ratio.

**Table 7:** Effect of Monensin (12 µg/ml) and Resveratrol (200 µg/ml) on volatile fatty acid molar proportion (EXP.3)

Substrate	Treatment ^1^	Acetic %	Propionic %	Butyric %	Isovaleric %	Valeric %	Total VFA(mM)	A/*P*^2^
Corn								
	CON	57.83	23.4^f^	16.5^ab^	1.19^ab^	1.11^a^	203.4	2.48^a^
	MON	55.75	32.2^b^	13.2^d^	1.1^bcd^	0.97^b^	187.3	1.73^e^
	RES	56.98	22.8^f^	17.8^a^	1.21^ab^	1.19^a^	197.9	2.50^a^
Wheat								
	CON	57.46	26.98^d^	13.6^bc^	1.15^bc^	0.78^dc^	197.1	2.19^c^
	MON	52.54	34.53^a^	11.3^dc^	1.04^cd^	0.61^e^	188.2	1.54^f^
	RES	57.83	26.5^ed^	13.7^bc^	1.30^a^	0.82^c^	202.1	2.13^c^
Barley								
	CON	60.03	25.1^e^	13.1^dc^	1.07^cd^	0.67^ed^	191.1	2.40^ab^
	MON	57.49	29.7^c^	11.4^dc^	0.84^e^	0.62^e^	198	1.95^d^
	RES	58.96	26.1^ed^	13.3^c^	0.99^d^	0.70^ecd^	189.4	2.27^bc^
SEM^3^		0.812	0.542	1.04	0.03	0.02	4.87	0.05
**Substrate effect**								
Corn		56.9^b^	26.1^b^	15.8^a^	1.16^a^	1.09^a^	196.2	2.24^a^
Wheat		55.9b	29.3^a^	12.9^b^	1.16^a^	0.74^b^	195.8	1.95^b^
Barley		58.8a	27^b^	12.6^a^	0.96^b^	0.66^c^	192.8	2.21^a^
**Treatment effect**								
	CON	58.4^a^	25.2^b^	14.4^b^	1.14^a^	1.11^b^	197.2	2.34^a^
	MON	55.3^b^	32.1^a^	11.95^c^	0.99^b^	0.97^c^	191.2	1.73^b^
	RES	57.9^a^	25.1^b^	14.9^a^	1.17^a^	1.19^a^	196.4	2.32^a^
SEM^3^		0.64	0.42	0.19	0.02	0.01	2.81	0.03
P-value								
Treatment		< 0.0001	< 0.001	< 0.001	< 0.001	< 0.001	0.271	< 0.001
Substrate		< 0.001	< 0.001	< 0.001	< 0.001	< 0.001	0.670	< 0.001
Treat×Sub		0.126	< 0.001	< 0.001	0.012	0.001	0.064	0.002

CON = control; MON = monensin; RES = resveratrol; represent average across 12 and 24 h (*n* = 6)

^2^ A/P acetate to propionate ration

^3^ SEM = standard error of the mean. *n* = 6 replicates / treatment

a-c Means in the same column for each substrate or effect with different superscripts differ (*P* < 0.05);

Effect of treatments, substrates, were significant (*P* < 0.001) for all parameters except for total VFA (*P* > 0.05), interaction between treatment and time (*P* < 0.001) for all parameter except for Acetic % and total VFA (*P* > 0.05)

No significant (*P* > 0.05) effect of time, time × substrate or time × treatment

## Discussion

The first experiment was conducted to examine the efficiency of different levels of RES, CUR and their MIX on preventing rumen acidosis in vitro compared to MON and CON. In order to evoke acidosis, higher concentration of glucose (4.5 g/45 ml) was used plus hay (0.1 g/45 ml) as substrate (Hutton et al. 2010). Based on Hutton et al. (2010), no buffer was used and the fermentation was allowed for only 6 h in order to identify the most effective treatment and concentration of the natural active component. The assay was found to be effective in simulating ruminal acidosis in vitro as the pH of CON declined to 5.13. MON was included as a positive control based on its established efficacy in preventing ruminal acidosis and its widespread use in ruminant feeds. Including MON allowed direct comparison between conventional ionophore-based control of lactic acid accumulation and phytogenic alternatives (Ahmed et al. 2022). MON was effective in controlling the decline of ruminal pH, however it reduced gas production compared to CON. MON disrupts the transmembrane gradients of Na⁺, H⁺, and K⁺ in ruminal microbes, which selectively inhibits gram-positive lactic acid-producing bacteria such as *Lactobacillus spp.* and *Streptococcus bovis*, while favoring the growth of gram-negative lactate-utilizing species such as *Megasphaera elsdenii* (Russell 1987). Such microbial modulation helps reduce lactic acid accumulation, stabilize ruminal pH, and enhance overall rumen fermentation efficiency.

At a concentration of 200 µg/mL, RES demonstrated efficacy comparable to MON in preventing ruminal pH decline. The polyphenolic structure of RES, a phytoalexin synthesized by certain plants, underlies its antimicrobial activity and role in plant defense responses (Ryu et al. 2022). The speculated mode of action of RES is due to the antimicrobial effects as a polyphenolic compound that could inhibit gram-positive bacteria. The lactic acid producing bacteria *Streptococcus bovis* and *Lactobacillus* spp. are classified as a gram-positive bacteria which could be sensitive to RES treatment. In contrary, *Megaspherea elsdenii* is a lactic acid-fermenting bacteria that are classified as a gram-negative bacteria could be enhanced with RES treatment.

CUR was included due to its known antimicrobial and anti-inflammatory properties. However, its lack of efficacy in preventing pH decline may be attributed to its limited bioavailability, likely resulting from intrinsic hydrophobicity, chemical instability, and susceptibility to photodegradation, as previously reported by Feng et al. (2020). The reason for the lack of CUR effect on pH could be also related to the fact that CUR could be utilized in fermentation by rumen microbes as evidenced by increased gas production and lowered pH values at lower concentrations (100 µg /ml) compared to CON. Utilization of CUR in fermentation by *Lactobacillus* bacteria was reported by Yong et al. (2019) can explain the lack of effect of CUR in preventing pH decline in the current study in both experiments 1 and 2. Both RES and CUR were not effective at a lower dose (100 µg /ml) when added individually. However, mixing them together at the same concentration (MIX 200; 100 µg /ml each) increased pH values, which could be evidence for an associative effect between both RES and CUR. While speculative, this associative effect may result from CUR-mediated changes in microbial membrane permeability, enhancing RES access to target bacteria. The mechanism of action of the associative effect of RES with CUR is not known and needs further studying.

The ability of RES and MIX to reduce lactic acid concentration and increase pH at 24 h of incubation of rumen fluid with buffer is an indication of their ability to control ruminal acidosis. Lactic acid is considered an intermediate product in rumen fermentation that should be fermented by specific microbes to produce VFA. However, high concentrations of highly fermentable carbohydrates cause the accumulation of lactic acid as a result of an imbalance between the production and utilization of lactic acid. Lactic acid is a strong organic acid that is ten time more potent than VFA because of the higher pKa; therefore, when it accumulates, it causes a rapid reduction in pH (Nagaraja et al. 1987). That explains the rapid decline of pH over time as a result of increased lactic acid production and accumulation in both the protection assay (EXP. 2) and the grain assay (EXP. 3) in the present study. Lower pH inhibited most gram negative bacteria, which include lactic acid-fermenting bacteria such as *Megaspherea elsdenii*. Moreover, lactic acid-producing bacteria like *Streptococcus bovis* and Lactobacillus spp. became the predominant bacteria at lower pH (Nagaraja et al. 1987).

In the protection assay (EXP. 2), increased propionate and valerate concentrations after RES treatments are probably due to an enhanced *Megaspherea elsdenii* bacteria population that is known to ferment lactic acid into propionate, acetate, and valerate through the acrylate pathway, which is known to produce more propionate compared to the succinate pathway (Nagaraja and Titgemeyer 2007). Lactic acid is fermented either through the succinate pathway by *Selenomonas ruminantum*, which ferments one unit of lactic acid to produce one unit of propionate, or by the acrylate pathway by *Megaspherea elsdenii*, which ferments three units of lactic acid to produce two units of propionate (Nagaraja and Titgemeyer 2007). Contrary to the result of the protection assay (EXP. 2), RES did not affect propionate production in the grain assay (EXP. 3).The inconsistency between the two experiments may be related to the different substrates used, which probably affected the population of microflora. The presence of hay (0.1 g/45 mL) in addition to glucose (4.5 g/45 mL) as a substrate in the protection assay (EXP. 2) may have promoted fibrolytic bacteria such as *Fibrobacter succinogens* and *Ruminococcus flavefaciens* that produce succinate, which could be fermented by *Selenomonas ruminantium* to produce propionic acid (Nagaraja and Titgemeyer 2007). This could explain the higher propionate-to-acetate ratio obtained by RES treatment in the protection assay (EXP. 2) but not in the grain assay (EXP. 3). Further, addition of RES increased total VFA compared to MON in the protection assay (EXP.2). However, neither RES nor MON affected total VFA production in the grain assay (EXP. 3) because of the different substrates used in each experiments. More research is needed to study the effect of RES on rumen microflora at different high-concentrate diets.

RES and MON maintained lower lactic acid concentrations below 20 mM only when corn was used as a substrate, not wheat or barley. This is in agreement with the finding of Fulton et al. (1979), who reported that a wheat-based diet caused a lower rumen pH than a corn-based diet. Although corn contains more starch than barley and wheat, the starch availability in corn is less than that in wheat and barley due to the protein structure surrounding starch granules in corn, which prevents starch availability for microbial fermentation (Benninghoff et al. 2015; Nagaraja and Lechtenberg 2007).

## Conclusion

This study demonstrates that RES, at concentrations of 200 and 400 µg/mL, mitigated in vitro-induced ruminal acidosis by increasing pH and reducing lactic acid accumulation. Its efficacy was comparable to MON, particularly when corn was used as the fermentable substrate. In contrast, CUR, at the same concentrations, had no significant effect on pH, lactic acid levels, or VFA profiles. RES enhanced propionic acid production and reduced the acetate-to-propionate ratio, while total VFA concentrations remained unchanged. These findings support the potential application of RES as a phytogenic alternative to MON in ruminant feeding programs, especially in corn-based diets prone to acidosis. However, in vivo studies are required to confirm its biological activity, safety, and efficacy under practical feeding conditions. Further research is also warranted to determine optimal inclusion rates, long-term effects, and interactions with dietary composition to support its application in commercial feed formulations.

## Data Availability

The datasets used and/or analyzed during the current study are available from the corresponding author on reasonable request.
